# Efficacy of a complex smartphone application for reducing hazardous alcohol consumption: Study protocol for a randomized controlled trial with analysis of in‐app user behavior in relation to outcome

**DOI:** 10.1002/mpr.1848

**Published:** 2020-09-08

**Authors:** Domonkos File, Beáta Bőthe, Máté Kapitány‐Fövény, Zsolt Demetrovics

**Affiliations:** ^1^ Institute of Psychology Eötvös Loránd University Budapest Hungary; ^2^ Département de Psychologie Université de Montréal Montreal Canada; ^3^ Department of Addiction Semmelweis University Faculty of Health Sciences Budapest Hungary; ^4^ Drug Outpatient Centre Nyírő Gyula National Institute of Psychiatry and Addictions Budapest Hungary

**Keywords:** alcohol intervention, digital intervention, online intervention, smartphone application, user behavior

## Abstract

**Objective:**

The efficacy of alcohol reduction applications is variable, and the underlying factors are largely unknown. The aim of this study is threefold: evaluate the relationship between user engagement and intervention efficacy, investigate the efficacy of the different functions applied, and investigate the efficacy of the intervention application compared to control groups.

**Methods:**

A randomized controlled trial will be conducted to determine the efficacy of a newly developed smartphone application compared to the controls in reducing alcohol consumption at a 30, 60, 90, 120, 150, and 180 days follow‐up. Hazardous drinkers, aged 18 years or older, will be recruited through web articles and will be randomized (blinded to their allocation), to receive one of the two versions of the application (educational or control application) for 30 days, or will be allocated to a wait‐list control group. Function usage times will be recorded on a single‐user level to determine the association between application usage and efficacy.

**Results:**

Data collection will be completed by July 2020, and follow‐up will be completed by January 2021.

**Conclusions:**

The evaluation of intervention efficacy as a function of user behavior will hopefully contribute to the science of developing more efficient alcohol intervention applications in the future.

**Abbreviations**
ANCOVA: analysis of covariance
AUDIT: Alcohol Use Disorders Identification Test
BCT: behavior change technique
DBCI: digital behavior change intervention
eBAC: estimated blood alcohol concentration
UK unit: United Kingdom unit (8 grams or 10 ml of pure alcohol)

## 1 INTRODUCTION

Excessive alcohol consumption is a significant public health problem (Brick, 2004) and a leading cause of death worldwide according to the latest statistics of the World Health Organisation (2014). Brief interventions delivered in health care settings to hazardous drinkers are effective (Kaner, 2010), but have limited reach due to lack of time, training, or financial resources (Heather, Dallolio, Hutchings, Kaner, & White, 2004; Kaner, 2010). In the past decade, there have been major advances in digital behavior change interventions (DBCIs) to help individuals with alcohol use disorders (Zhang, Ward, Ying, Pan, & Ho, 2016).

As Crane (2017) summed up, nine out of 10 meta‐analyses or systematic reviews of DBCIs concluded (Carey et al., 2009, 2013; Dedert et al., 2015; Donoghue, Patton, Phillips, Deluca, & Drummond, 2014; Kaner et al., 2017; Khadjesari, Murray, Hewitt, Hartley, & Godfrey, 2011; Riper et al., 2011, 2014; White et al., 2010) that DBCIs reduce the amount of alcohol consumed, the frequency with which it was consumed and/or problems related to its consumption, relative to control conditions (Crane, 2017). Effect sizes in DBCIs were mostly small (ranging from *d* = 0.20 to *d* = 0.42) and indicated a mean reduction in consumption of approximately 24 g of alcohol, or 3 UK units of alcohol per week (range 12–33 g; Crane, 2017). It is important to note that the aforementioned DBCIs have been provided on the web. Although smartphone applications have the potential to help people manage their behavior in various health‐related fields, like physical activity, smoking or healthy diet (Bort‐Roig, Gilson, Puig‐Ribera, Contreras, & Trost, 2014; Bricker et al., 2014; Coughlin et al., 2016, respectively), there is limited evidence for the effectiveness of applications aiming to foster safe alcohol consumption (Garnett, Crane, Michie, Wesr, & Brown, 2016). According to Attwood et al.'s (2017) results, engaged users showed a significant reduction in alcohol consumption after the first week of application usage (mean −4.9 UK units), with no further improvement over time (week four mean reduction was −3 UK units). Gonzalez and Dulin (2015) found a reduction in hazardous drinking days among alcohol‐dependent users over a 6‐week trial compared to a bibliotherapy group. Gajecki et al. (2017) found a skill training smartphone app to be successful in reducing the likelihood of excessive alcohol consumption among excessively drinking university students both at 6 and 12 weeks post‐intervention. Also, they reported a reduction in the quantity of drinking at 6 weeks post‐intervention and a reduction in the frequency of drinking both at 6 and 12 weeks post‐intervention. To conclude, studies investigating smartphone applications that aim to reduce the consumption of alcohol reported positive outcomes, with varying effectiveness.

A major issue in application development is engaging users with the application content and features, reflected by the high drop‐off rates; 95% of applications are not used longer than a month (Milward et al., 2016). To date, relatively few studies were conducted to explore user preferences in relation to alcohol consumption reduction applications (Attwood, Parke, Larsen, & Morton, 2017). Earlier studies relied on the user ratings of publicly available applications (Crane, Garnett, Brown, West, & Michie, 2015), or on the evaluation of targeted sample groups (Gajecki, Berman, Sinadinovic, Rosendahl, & Andersson, 2014; Milward et al., 2016). The findings of these studies indicate that user preferences include personalized content, social networking capability, and ease of functionality, especially in terms of entering drinking data (Attwood et al., 2017). Zhang et al. (2016) reported that notifications and information sharing were the most useful components, while psychotherapeutic aspects (including a functional analysis chart and a behavioral goals chart) were the least useful feature of their mobile application, based on user self‐reports. Crane, Garnett, Michie, West, and Brown (2018), in a factorial design study, found that the combination of normative‐feedback and cognitive bias retraining and self‐monitoring and feedback and action planning yielded improvements in alcohol‐related outcomes after 4 weeks. Attwood et al. (2017) analyzed the data of the large user base of a publicly available application (Drinkaware), completed with user interviews, and a common preference was expressed for more personalized content (Attwood et al., 2017).

Although the above studies have a great contribution to our understanding of user preferences in mobile applications reducing alcohol consumption, the link between application usage and efficacy is not fully understood yet. According to Milward, Deluca, Drummond, and Kimergård (2018), user engagement—that is, how long and how often the participant uses the intervention (Crutzen et al., 2011)—is a significant predictor of intervention efficacy. In case of low user engagement, an application is unable to promote behavioral changing progress (Fitzgerald & McClelland, 2017), thus exploring the correlation between user engagement and intervention efficacy to reduce harmful alcohol consumption is a key research objective.

To investigate this question, a new smartphone alcohol intervention application was developed—Yoozan—with built‐in timers recording usage times of the different functions separately for every user. A randomized controlled trial will be conducted, where participants will use Yoozan or a control application (with restricted functionality, described in Methods) for 30 days, or will be allocated to a wait‐list control group. By analyzing usage times and the efficacy of the application in reducing the amount of alcohol consumed, the primary aim of the study is to evaluate the relationship between user engagement and intervention efficacy. The secondary aim of the study is to investigate the efficacy of the application functions by investigating the relationship between usage times of the application functions and intervention efficacy. The tertiary aim of the study is to investigate the efficacy of the intervention application compared to an active control application and to a wait‐list control group.

We hypothesize that there will be a positive correlation between engagement (reflected in usage time) and between the extent of reduction in alcohol consumption. We also hypothesize that Yoozan users will consume less alcohol at post‐intervention and 30, 60, 90, 120, 150, and 180 days follow‐up, compared to the participants in the control conditions.

## 2 METHODS

### 2.1 Study design

**FIGURE 1:**
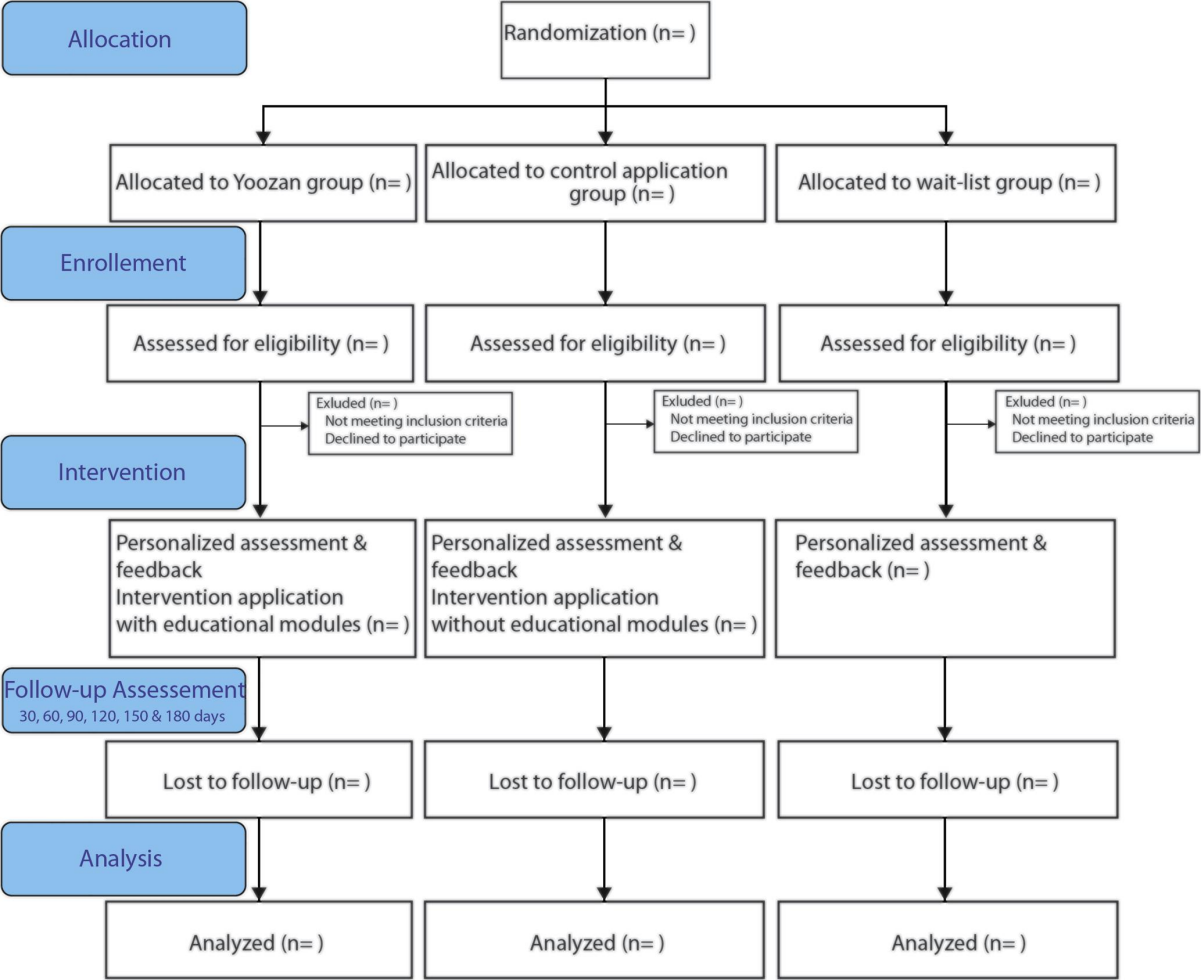
Overview of study design according to CONSORT format

The aforementioned aims will be achieved in a randomized controlled trial with three parallel arms. Two smartphone application‐based interventions will be compared: a new intervention for reducing harmful drinking (Yoozan) and a control application (Yoozan without educational modules). Assessments will be completed at baseline, post‐assessment (30 days from registration), and follow‐ups at 60, 90, 120, 150, and 180 days (see Figure 1). The study was approved by the Hungarian Ethics Committee (2019/293). Consent is obtained electronically from all participants. The study is conducted in accordance with the Declaration of Helsinki.

### 2.2 Recruitment of study participants and trial flow

Recruitment took place from December 2019 and is currently ongoing. Participants are recruited through promotion articles published on Hungarian Internet portals. In the articles, Yoozan is introduced with a call to participate in testing in case the reader wants to change his/her drinking habits. All interested participants are directed to the webpage of the study, from where the application can be downloaded. Based on a block randomization method, an equal number of participants download either Yoozan, the control application, or allocated to the wait‐list control group. After installing the application, participants are asked to read the participant information sheet and provide informed consent and a valid email address for which usability questionnaire and follow‐ups will be sent (see Figure 1).

Participants allocated to the wait‐list group are informed that 6 months later, the application will be sent to their email addresses and are encouraged to fill a few tests (7‐day Timeline Followback, Alcohol Use Disorders Identification Test [AUDIT]) with instant feedback.

Study inclusion criteria are informed consent via the Web form to ensure knowledge of procedures and the declaration of consent; participant age ≥18 years, to ensure a minimal age of participation; ownership of an Android‐operating smartphone and at least weekly Internet access, to ensure program access; a screening AUDIT score ≥8 out of 40, to include adults with potentially hazardous or harmful alcohol consumption; and an indication of an attempt to reduce alcohol consumption (screening motivation score ≥2 out of 5), to ensure a minimal willingness to change. Exclusion criterion is participation in other treatments for the reduction of alcohol consumption, to avoid confounding treatment effects.

The study targets Hungarian speaking users; thus, the language of the intervention is Hungarian. If the intervention is effective, translation to English and upload to App Store and Google Play is planned.

### 2.3 Sample size calculation

Based on previous studies, realistic anticipation is a Cohen *d* of 0.20 for the effect size differences between the two study arms. To demonstrate standardized effect sizes, Cohens *d* of 0.20 with power (1−β) of 80% and an alpha of 0.05 in a two‐sided test performed with G*Power software (Faul, Erdfelder, Buchner, & Lang, 2009) 394 individuals would need to be included in each group. Based on the attrition rate of a self‐directed treatment‐seeker sample of Crane et al. (2018), we expect that 27% of study participants will respond to the first follow‐up, increasing the initial sample required by 3180; thus, we aim to recruit a total of 4356 participants.

### 2.4 Measures

At baseline weekly alcohol consumption, AUDIT score, motivation to change drinking habits, and sociodemographic data (age and gender) are assessed. Weekly alcohol consumption is assessed by the 7‐day Timeline Followback (e.g., see Sobell & Sobell, 1992) by users entering the consumption for each day of the previous week from a predefined list of drinks. It allows defining consumption measures that hopefully contribute better comparability between studies (Shorter et al., 2019), such as the frequency of drinking and heavy drinking (on a weekly level) and the summed consumption in standardized units. The AUDIT is a validated 10‐item screening tool developed by the World Health Organization (WHO) to assess alcohol consumption, drinking behaviors, and alcohol‐related problems. It is particularly designed for health care practitioners, but with suitable instructions, it can be self‐administered (Babor, Higgins‐Biddle, Saunders, & Monteiro, 2001). Motivation to change drinking habits is measured with the question “How important is it for you to change your drinking habits?” with five options to answer (1: not important, 5: very important).

The follow‐up questionnaires consist of the 7‐day Timeline Followback and application usability test. Subjective rating of application functions is measured at the first (30 days) follow‐up, with questions regarding the perceived effectiveness of each function helping to reach alcohol‐related goals, with five options to answer (1: very poor, 5: very helpful).

Besides the self‐administered tests, usage times of the application are recorded, and the following variables are formed: total usage, frequency (the number of usage sessions), and usage time of functions.

## 3 INTERVENTIONS

### 3.1 Educational application: Yoozan

The educational application, Yoozan, is a newly created smartphone application‐based intervention centered on Behavior Change Techniques (BCTs). BCTs are observable, replicable, and an irreducible component of an intervention designed to alter or redirect causal processes that regulate behavior (Michie et al., 2013). The motive during the selection of tools was to implement evidence‐based techniques to a mobile interface. Our work was not a pioneer in this respect; therefore, a relatively large body of evidence was available. The Drink Less application introduced by Garnett et al. (2016) used well‐reasoned modules in concordance with others (e.g., Attwood et al., 2017; Crane, 2017; Dulin, Gonzalez, King, Giroux, & Bacon, 2013), so we followed their work regarding the theoretical assumptions of the used functions. These are goal‐setting, self‐monitoring and feedback, action planning, normative feedback, and identity change (see Garnett et al., 2016). Based on the conclusion of Webb, Joseph, Yardly, and Michie (2010), by which the more BCTs were used in Internet‐based interventions, the more effective they were, additional functions were added to Yoozan. These are a chat room, providing peer support, evaluation of progress through achievements, and a state/mood‐activity diary. The state/mood‐activity diary was based on an extremely popular and well‐rated application, Daylio (Habitics, 2020; more than 5 million installs on Android, with an overall rate of 4.7 out of 5 based on more than 250,000 votes in 2020, Q2), the motive behind its use was twofold. On the one hand, based on the outstanding popularity of this formula, we anticipated that this tool would strengthen user engagement. Also, the state/mood‐activity data‐entry interface was developed in a way that it was only available once a user was done tracking the consumed drinks, serving as an additional motivation factor for tracking drinks. On the other hand, we assumed that tracking moods and activities might serve as valuable feedback, especially in relation to alcohol consumption.

The selection of tracked states was determined by states/moods known to be influenced by excessive alcohol consumption. A decrement in attention‐related cognitive abilities and memory functions are well documented in case of a hangover (e.g., Anderson & Dawson, 1999; McKinney & Coyle, 2004), thus a tracked state is (1) *intellectual sharpness*. Fatigue and a poor sense of well‐being are symptoms of a hangover (Wiese, Shlipak, & Browner, 2000), which is manifested in the state of (2) *energy level* and (3) *overall rating of the day*. Also, acute stress may produce an increase in alcohol consumption (de Wit, Söderpalm, Nikolayev, & Young, 2006), thus tracking (4) *stress level* in relation to alcohol consumption might help users to recognize a potential trigger of drinking. Also, Andersson, Söderpalm, and Berglund (2007) reported a positive relationship between reported stress reduction and the amount of alcohol consumed.

**FIGURE 2:**
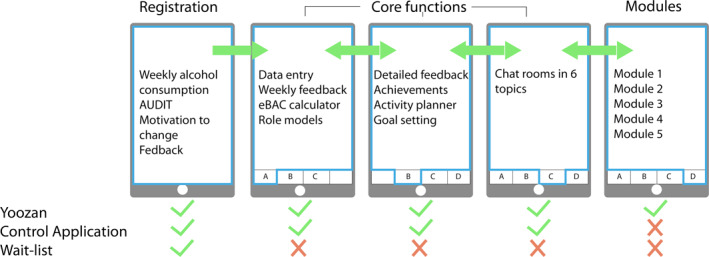
An overview of the functions and navigation between functions

The application consists of three functional units: (1) registration and feedback, (2) core functions, and (3) educational modules (see Table 1 and Figure 2 for an overview).

**TABLE 1:** An overview of the functions

Functional unit	Function	Rationale
Registration (present in Yoozan, control application and wait‐list groups)	Baseline tests (weekly alcohol consumption, AUDIT, and motivation to change)	Set pre‐intervention baseline
	Personalized feedback (weekly alcohol consumption and AUDIT)	Presenting drinking profile and normative comparison, as effective tools for reducing harmful alcohol consumption (Miller et al., 2013)
Core functions (present in Yoozan and control application groups)	Drink, activity, and mood diary	Providing a platform for clear, organized tracking of alcohol related data
	eBAC calculator	Calculating and displaying the estimated blood alcohol level
	Achievements	Creating goal commitment and providing additional feedback
	Role models	Promoting non‐problem drinking, harm reduction or abstinence
	Activity planner	Encouraging alternative activities other than alcohol consumption
	Community chat	Providing peer support
Modules (present only in Yoozan)	Module 1—consequences of excessive alcohol consumption and goal setting	Facilitating conscious evaluation of the effects of alcohol on ones' life
	Module 2—facilitate action planning and problem‐solving	Presenting a problem‐solving technique with practical information on how to prevent excessive alcohol intake
	Module 3—identify contextual factors and self‐control practices	Facilitating conscious evaluation of contextual factors related to excessive alcohol consumption
	Module 4—refusal skills training	Presenting communication techniques to help successful refusal of alcohol
	Module 5—relapse management	Presenting warning signs of relapse, action plans, and general information

Abbreviations: AUDIT, Alcohol Use Disorders Identification Test; eBAC, estimated blood alcohol concentration.

### 3.2 Registration and feedback

The aim of the registration process is to collect baseline measures (weekly alcohol consumption, AUDIT, and motivation to change, see Table 2) and to provide feedback on the alcohol use of the participants.

**TABLE 2:** Overview of the measurements

Measures	Baseline (t0)	Day 30 (t1)	180 days follow‐up (t2, t3, … t6)
Demographic (age and gender)	X	‐	‐
Demographic (level of education and place of residence)	‐	X	‐
7‐day Timeline Followback (Sobell & Sobell, 1992)	X	X	X
AUDIT (Babor et al., 2001). World Health Organization	X	‐	‐
Motivation to change	X	‐	‐
Usability ratings of the application	‐	X^a^	‐

^a^ Excluded in wait‐list control group.

Abbreviation: AUDIT, Alcohol Use Disorders Identification Test.

### 3.3 Core functions of the application

Core functions include self‐monitoring (drink, mood, and activity tracking and feedback), estimated blood alcohol concentration (eBAC) calculator, role models, evaluation of progress (achievements), activity planner, goal‐setting, and community chat.

**FIGURE 3:**
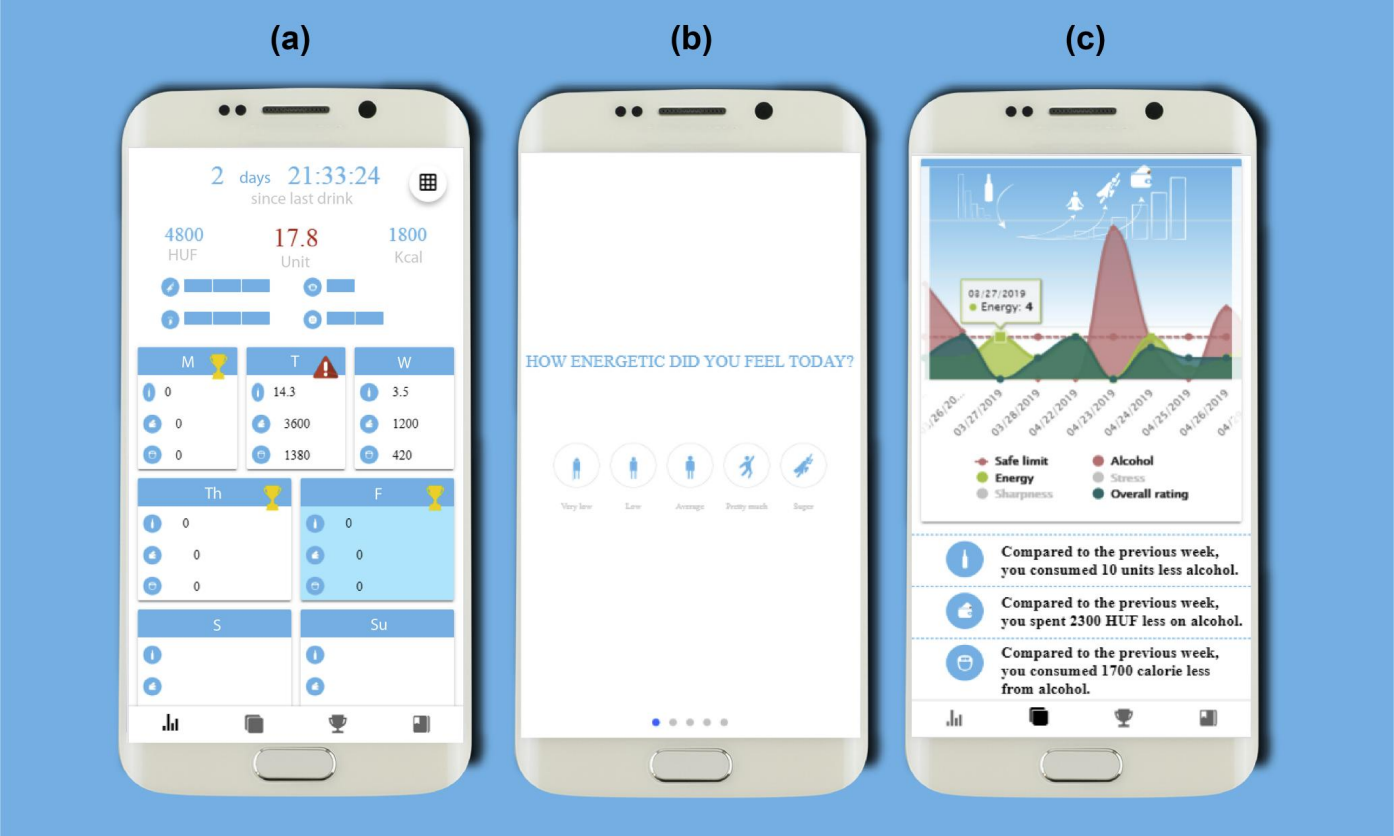
Screenshots of the application: (a) data entry, (b) state/mood diary data entry, and (c) feedback of the tracked moods and alcohol consumption (translated from Hungarian to English for publication purposes only)

The *self‐monitoring function* can be divided into two functional units; data entry and feedback. Modern research on motivation reveals that persistence increases with the subjective proximity to goal attainment (Cheema & Bagchi, 2011; Louro, Pieters, & Zeelenberg, 2007), so we aimed to break the long process of cutting back or quit drinking into reasonable and achievable goals. Since health guidelines define the quantity of alcohol intake in weekly periods, the application was designed to focus users on this interval. According to this, the data entry interface is a modified calendar showing the current week and drinking‐related data of the user. The aim of the feedback page is to provide detailed information about the users' drinking habits, mood, and activities. Data is shown on various graphs, with the option to select and filter, to ensure in‐depth exploration (see Figure 3).

The *eBAC calculator* is a form of personalized feedback, an integral part of the Alcohol Skills Training Program (Fromme et al., 1994), calculating and displaying the estimated blood alcohol level of the individual, the excepted time it reaches zero and whether the user is allowed to drive.

Social control and social learning theories both emphasize the importance of *role models* in promoting non‐problem drinking, harm reduction, or abstinence (Moos, 2008). The role models function is a popup window appearing after opening the application, introducing a well‐known person (actor or singer), who successfully overcome his/her addiction.

Since gamification increases user activity (Juho, 2015), evaluation of progress is expressed through *badges, achievements, and trophies* to increase self‐efficacy and satisfaction, to create goal commitment, and to provide additional feedback. Achievements are divided into (1) *community achievements* where the summed outcomes (change in alcohol consumption, saved money, and avoided calories) of all users are displayed, and (2) *individual achievements*, which reflect the progress and goals of the individual user.

The idea behind the *activity planner* is that people tend to drink less during activities typically does not involve alcohol, for example, participation in volunteering is associated with less alcohol use (Weitzman & Kawachi, 2000). In Module 3, users could select the time periods they were engaged in heavy drinking, which are feedbacked as risky periods in the activity planner interface. Users are encouraged to set programs on those periods, either by adding their own ideas or by using a random activity generator.

The aim of the goal‐setting function is to facilitate goal statements regarding regulating alcohol consumption. Goal setting is an effective component of alcohol interventions (Tanner‐Smith & Lipsey, 2015), and the ability to set own drinking goals is highly valued by users (Giroux, Bacon, King, Dulin, & Gonzalez, 2014).

The aim of the *community chat* is to provide peer support. Peer support—the process of giving and receiving nonprofessional assistance from individuals with similar conditions—(Tracy & Wallace, 2016), is a key component of many existing addiction treatment approaches, such as the 12‐step programs (Allen, Anton, Babor, & Carbonari, 1997) or the community reinforcement approach (Meyers, Miller, Smith, & Tonigan, 2002). Online support groups also offer peer support, motivation, and positive role‐modeling (Moos, 2007). Based on Urbanoski, van Mierlo, and Cunningham (2016), relatively low activity is expected from most members but having an option for social networking might be a potential way to reduce attrition (Bennett & Glasgow, 2009). Community chat has six chat rooms with different topics: (1) successes, (2) lifestyle, (3), techniques (4), urgent (about to slip), (5) relapse, and (6) free. The rationale behind divide the chat into topics was to facilitate goal‐oriented communication between users.

### 3.4 The content of the educational modules

The aim of the modules is to provide the user all relevant information and help them to find a way to commit to the decision they have made and link the change to the user's broader goals and values, with a lack of coercion or direct persuasion from the application, as suggested by Resnicow and McMaster (2012).

The goal of *Module 1—consequences of excessive alcohol consumption and goal setting*—is to urge the user to evaluate the positive and negative consequences of his or her alcohol consumption present and in the future, to present the positive aspects of controlled drinking or abstinence through general descriptions and personal experiences and to set goals regarding alcohol consumption. There are two goal types, either aiming to reduce consumption or total abstinence for a period of time specified by the user. At the end of every module, users have the option to modify their goals.

*Module 2—facilitate action planning and problem‐solving*—is started with personalized feedback reflecting the positive consequences of alcohol perceived by the user and alternatives for replacing them. It is followed by an introduction of the monetary consequences of cutting back derived from the baseline consumption and the goals set in Module 1. After that, the WOOP technique is presented as a tool for action planning. The WOOP technique is an imaginary exercise that increases goal commitment and behavioral change through performing four sequential steps; (1) identifying a meaningful goal (wish); (2) identifying and imagining the best outcome of accomplishing this goal (outcome); (3) identifying and imagining the critical inner obstacle to accomplishing that goal (obstacle); and (4) forming an “if‐then” plan to overcome that obstacle (Saddawi‐Konefka et al., 2017). Also, tips/techniques are presented in three domains; lifestyle changes, techniques for reducing alcohol consumption, and techniques in case of cravings.

*Module 3—identify contextual factors and self‐control practices*—is designed to help users identify risky contextual (where, when, whom) factors, with the option to set specific goals regarding conscious avoidance of risky situations. Marlatt and Donovan (2005) highlight the importance of geographical areas represent a high‐risk situation for relapse, and encourage people to avoid them as much as possible. Module 3 ends with self‐control practices with personal experiences of former heavy drinkers.

*Module 4—refusal skills training*—consists of communication techniques targeting successful refuse of alcoholic drinks and interactive situations for practice. This module helps users to recognize social pressure to drink, and prepare them with lined up strategies to refuse drinks in a confident, yet appropriate way.

*Module 5—relapse management*—covers the exploration of warning signs of relapse, action plans, and general information. The aim of this module is to inform users that temporary relapses might happen, which should not reduce their motivation to achieve their goals. Presenting personal experiences of former hazardous drinkers, users are encouraged to view their consumption goals as a long‐term process, with the possibility to learn from it.

At the final stage of application development, a small group of users (*n* = 8) were involved in app testing. Based on their feedback regarding ease of use, language‐wording, and bugs, the application was modified.

### 3.5 Control application

The control application has the same core functions as Yoozan, without the educational modules. With the exception of WHO alcohol consumption guidelines, core functions do not contain explicit information on alcohol use. Thus, the rationale behind applying a control application was to investigate the effectiveness of the core functions in reducing alcohol consumption, without providing information on alcohol‐related topics.

### 3.6 Wait‐list control group

7‐day Timeline Followback and AUDIT are assessed, and personalized feedback is given for participants willing to participate in the study. Participants in the wait‐list control group receive access to Yoozan 180 days later.

## 4 RESULTS

The composition of our sample will be demonstrated via descriptive statistics of participants' age, gender, and baseline 7‐day Timeline Followback, AUDIT measures, and motivation to change scores. Random imbalances between the intervention and control groups will be examined using chi‐squared tests. To evaluate the efficacy of the intervention, a repeated measure one‐way between‐groups analysis of covariance (ANCOVA) will be conducted. The analysis model will be built with group allocation as the independent variable, change in alcohol consumption as the dependent variables (the difference between baseline and follow‐ups 7‐day Timeline Followback in consumed alcohol units, binge drinking occasions, and drinking frequency), and age, sex, and motivation to change as covariates. Prior to the analysis, we will make sure that the specific assumptions for normality and homogeneity of variance for the one‐way ANCOVA will be met. In situations where data is missing—for example, with participants who are lost to the 6‐month follow‐up—the mixed model approach will be used to estimate missing values.

Multilevel modeling will be used to estimate the relationship between change in alcohol consumption (consumed alcohol units, binge drinking, and drinking frequency), application usage (total usage time, usage frequency, and usage time of functions) and application usability ratings in the educational and control application groups.

## 5 DISCUSSION

This study protocol describes the design of a randomized controlled trial with the following aims: (1) evaluate the relationship between user engagement and intervention efficacy, (2) investigate the efficacy of the application functions by investigating the relationship between usage time of the application functions and intervention efficacy, and (3) investigate the efficacy of the educational application compared to an active control application and to a wait‐list control group. To the best of our knowledge, this will be the first study to take behavioral data into account in the evaluation of the effectiveness of a complex alcohol reduction application on a large sample. Crane et al. (2018) recorded usage times in a factorial design study, with no significant differences between application components. They also found that application components in combinations led to improvements in alcohol‐related outcomes (Crane et al., 2018), supporting the conclusion of Webb et al. (2010), that effectiveness increases with the number of BCTs. Zhang et al. (2016) found in their self‐report study that notifications and information sharing were the most, while psychotherapy was the least useful feature of their mobile application. Attwood et al. (2017) observed mixed feedback regarding the usefulness of the individual features, which emphasizes the heterogeneity of user needs.

With an attempt of taking the continuously growing knowledge of the field into account, we developed a mobile application, Yoozan, with a wide range of functionality: self‐monitoring and feedback; action planning; normative feedback; identity change; community support; goal setting; evaluation of progress; and mood‐diary. We assume that functions/components in a complex application will compete for usage, resulting in different usage times in an ecologically valid context. By registering usage time of functions on the single‐user level, it is possible to examine application efficacy as a function of usage habits such as usage time, usage frequency, the number of used functions, and usage time of functions. This data will serve as useful guidance for future application development, helping to specify effective functions and user behaviors associated with high efficacy.

The following limitations of the protocol must be considered: (1) all measurements except behavioral data will be self‐reported that may be prone to biases (e.g., social desirability bias), (2) possible unequal group sizes leading to reduced statistical power due to the randomization process occurring before assessment for eligibility, (3) potentially there will be missing follow‐up data of the dropped outs, and (4) the application only available on Android operating smartphones.

Although mobile phones offer the potential to reduce the harms of hazardous drinking (Crane et al., 2015), up to date there have been relatively few studies conducted that evaluate the efficacy of evidence‐based smartphone applications aiming to reduce alcohol consumption (e.g., Crane et al., 2018; Gonzalez & Dulin, 2015; Zhang et al., 2016). By applying a randomized controlled trial, the results of the present study will be comparable with previous studies and will be informative about the overall efficacy of alcohol intervention applications. By investigating the behavioral correlates of the application, we hope that a more complex understanding of application efficacy will be achieved, making more focused intervention development possible in the future.

## CONFLICT OF INTEREST

There are no conflict of interest.
